# Supplementary figures and images for: Development and validation of a subjective end-of-life health literacy scale
Source: PLoS One. 2023 Oct 13;18(10):e0292367. doi: 10.1371/journal.pone.0292367 (PMC10575492; doi:10.1371/journal.pone.0292367)

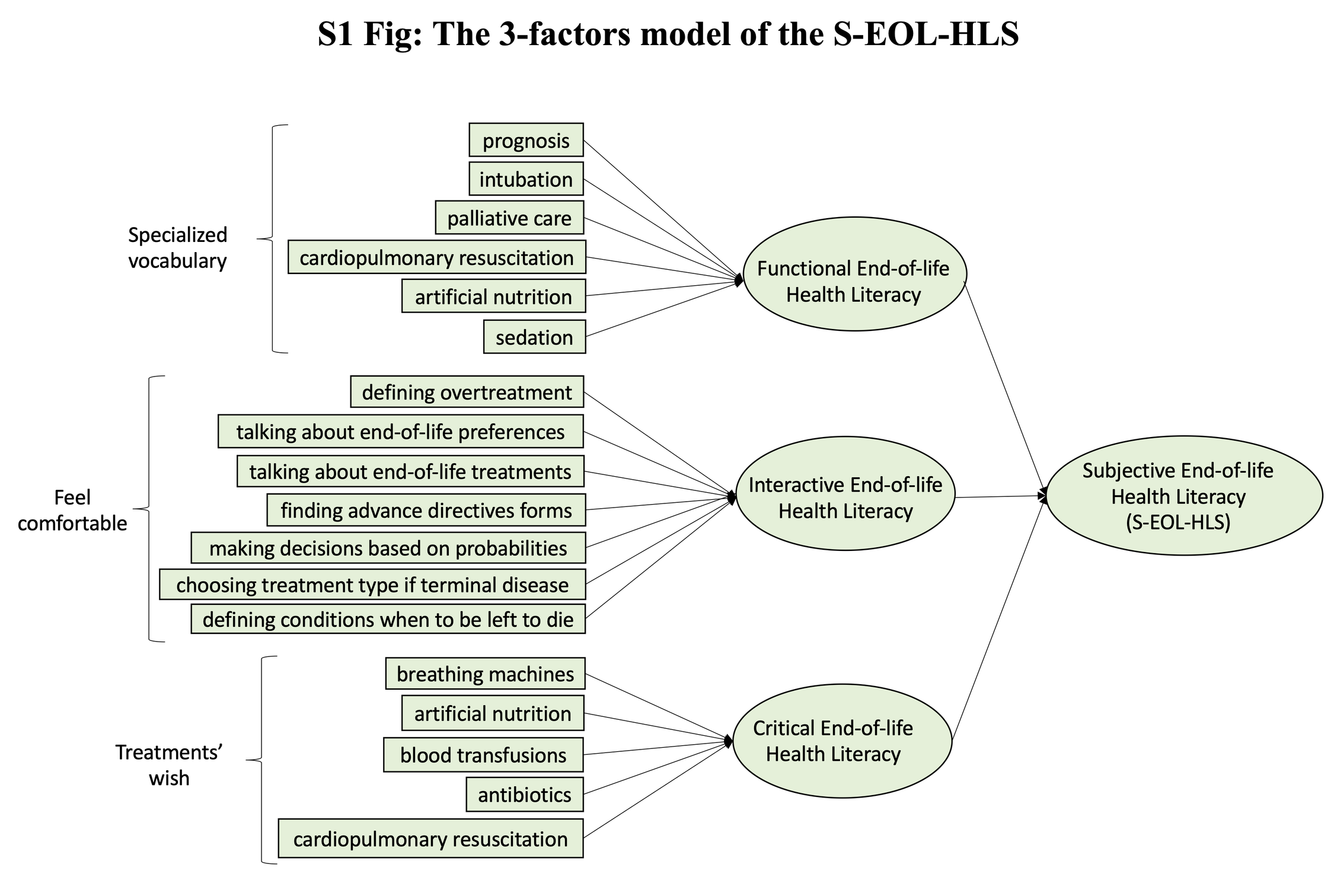

Supplement: S1 Fig — (TIF) [file pone.0292367.s001.tif]

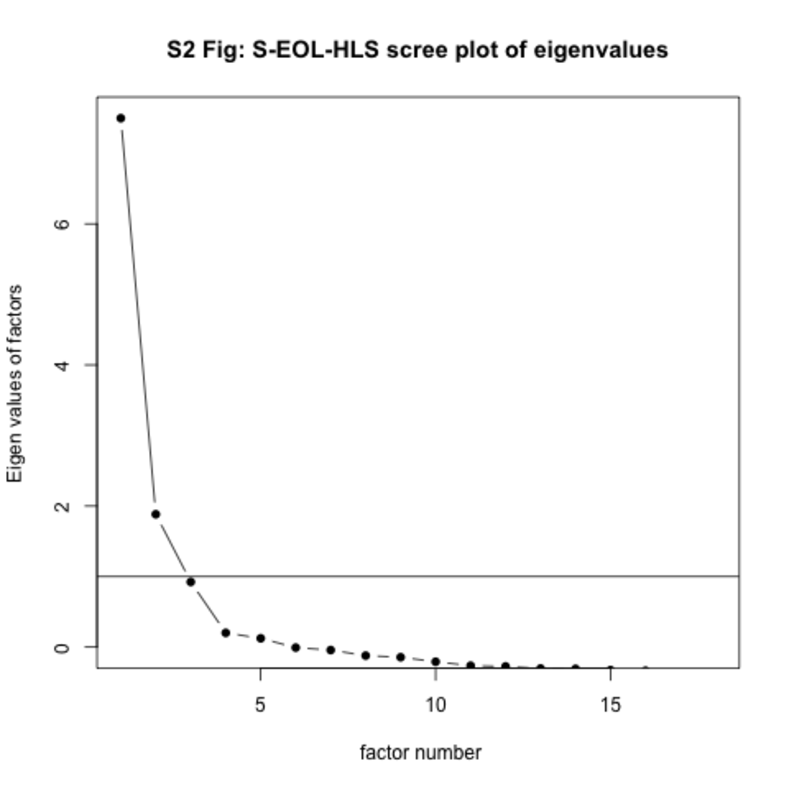

Supplement: S2 Fig — (TIF) [file pone.0292367.s002.tif]
