# Supplementary material for: Development and validation of a subjective end-of-life health literacy scale
Source: PLoS One. 2023 Oct 13;18(10):e0292367. doi: 10.1371/journal.pone.0292367 (PMC10575492; doi:10.1371/journal.pone.0292367)
Supplement: S2 Table — (DOCX) [file pone.0292367.s004.docx]

| **S2 Table: The 16 items from the HLS-EU-Q16 scale:** |
| --- |
| First, we would like to ask you how comfortable you feel when dealing with health-related information.  For you, how easy or difficult is it to…  Answer categories: "Very easy", "Fairly easy", "Fairly difficult", "Very difficult"   1. Understand your doctor's or pharmacist's instructions on how to take a prescribed medicine? 2. Follow instructions from your doctor or pharmacist? 3. Understand what doctor says to you? 4. Find out where to get professional help when you are ill? 5. Find information on treatments of illnesses that concern you? 6. Use the information the doctor gives you to make decisions about your illness? 7. Judge when you may need to get a second opinion from another doctor? 8. Understand health warnings about behaviour such as smoking, low physical activity, and drinking too much? 9. Understand why you need health screenings? 10. Find information on how to manage mental health problems like stress or depression? 11. Decide how you can protect yourself from illness based on information in the media? 12. Judge if the information on health risks in the media is reliable? 13. Understand advice on health from family members or friends? 14. Judge which everyday behaviour is related to your health? 15. Find out about activities that are good for your mental well-being? 16. Understand information in the media on how to get healthier? |
